# Using digital technology to ensure food supply stability: a case study of achieving food security in China

**DOI:** 10.3389/fnut.2026.1880467

**Published:** 2026-07-02

**Authors:** Danqi Wei

**Affiliations:** School of Finance and Economics, Jimei University, Xiamen, China

**Keywords:** agricultural socialized services, climate risk, digital technology, food security, land transfer, supply of food

## Abstract

**Background:**

Under the combined pressures of frequent extreme climate events and the spread of unilateral trade policies, global agricultural industry and supply chains are facing an unprecedented crisis. Digital technology (DT) is a key driver of the new technological revolution and is profoundly reshaping food production, distribution, and consumption. However, its comprehensive impact on food security and the underlying mechanisms remain unclear.

**Methods:**

This study systematically elaborates the theoretical logic through which digital technology enhances food security. Using panel data from 285 prefecture-level cities in China from 2011 to 2024, we construct an innovative urban DT composite index based on the number of artificial intelligence patents and the coverage rate of digital infrastructure. A dynamic spatial Durbin model and a mediation effects model are employed for empirical testing.

**Results:**

The findings reveal three main results. First, DT has a significant direct positive effect on urban food security and a significant positive spatial spillover effect, meaning that local DT development improves food security in neighboring regions. These results remain robust after using instrumental variable methods, substituting core variables, and performing multiple robustness checks. Second, the mechanism analysis shows that DT indirectly enhances food security by improving agricultural socialized services, accelerating land transfer efficiency, and reducing climate-induced volatility in agricultural production. Among these channels, agricultural socialized services have the strongest mediating effect. Third, heterogeneity analysis indicates that the enabling effect of DT on food security is more pronounced in non-major grain-producing areas, regions with flat terrain, and cities with higher levels of financial development, suggesting that DT helps bridge the digital divide in agricultural development across regions.

**Conclusion:**

This study deepens the understanding of food security mechanisms in the digital economy era and provides a solid theoretical and empirical foundation for formulating precise, differentiated policies for digital villages and smart agriculture, thereby supporting the development of new-quality productivity in agriculture.

## 1 Introduction

Food security serves as a fundamental pillar of national security, underpinning economic development, social stability, and public welfare. The world is currently undergoing profound changes unseen in a century. A confluence of challenges, including the increasing frequency of extreme weather events, the pronounced vulnerability of international agricultural supply chains, and intensifying resource and environmental constraints, is posing severe tests to food security systems across countries (1, 2). In this context, a critical global issue has emerged: how to harness the outcomes of the new technological revolution, particularly digital technologies such as artificial intelligence, big data, and the Internet of Things, to enhance agricultural productivity, optimize resource allocation, strengthen system resilience, and thereby consolidate national food security. Digital technologies (DT) are penetrating all aspects of agricultural production, operation, management, and services with unprecedented breadth and depth, giving rise to new forms and models such as precision agriculture and smart agriculture. These developments create new historical opportunities to overcome traditional constraints on agricultural development (3, 4, 59). Nevertheless, key questions remain: what specific pathways enable digital technology to empower food security? Do its effects exhibit spatial disparities and regional heterogeneity? Answering these questions is of crucial theoretical and policy significance for promoting the modernization of agriculture and achieving a high level of food security.

The existing literature has extensively explored the relationship between digital technology and agricultural development. Several studies have focused on the role of digital technology in enhancing agricultural productivity. For instance, scholars have found that digital inclusive finance can increase total factor productivity in agriculture by easing farmers' credit constraints and promoting their adoption of modern agricultural technologies (5, 61). Similarly, the construction of digital villages has stimulated farmers' entrepreneurial vitality and optimized rural industrial structures through information and resource effects (6, 7). Another strand of research has examined the socioeconomic effects of digital technology (DT) at the macro level, including its role in promoting green and low-carbon transformation (8–11), enhancing urban resilience (12–14), and posing challenges to jobs and content (15–18). These studies provide rich perspectives for understanding the enabling role of digital technology. However, systematic research specifically targeting the core issue of digital technology and food security remains insufficient, particularly regarding in-depth exploration of its mediating mechanisms and comprehensive consideration of spatial dimensions. Existing studies often treat the impact of digital technology as an opaque process, failing to clearly reveal how it ensures the stability and sufficiency of food supply through specific changes in agricultural production relations and factor allocation.

Specifically, several areas in the current research warrant further development. First, regarding the identification of mechanisms, the existing literature largely explores the issue from a single dimension, such as macro-level industrial structure upgrading or micro-level changes in farmers' behavior. It rarely links digital technology (DT) to fundamental changes in the organization of agricultural production. The improvement of agricultural socialized service systems, the large-scale transfer of land resources, and the adaptation to increasingly severe climate risks represent three key leverage points in modern agricultural development. These are also areas where digital technology can best capitalize on its advantages in information matching, intelligent decision-making, and risk early warning. Adopting these three factors as core mediating variables can more accurately delineate the internal logical chain through which digital technology enhances food security. Second, concerning the measurement of core variables, the quantification of DT mostly relies on digital inclusive finance indices or general Internet penetration rates, which only partially capture the essence of digital technology. The development of DT encompasses not only financial inclusiveness but, more crucially, hard-core technological innovation and application centered on artificial intelligence and big data. Therefore, constructing a comprehensive index that reflects both cutting-edge technological innovations and widespread infrastructure is essential for accurately assessing the impact of DT. Third, with respect to research methods, there is a significant spatial correlation between food security and DT development. Agricultural production in a given region is influenced not only by local factors but also by technology diffusion, market linkages, and spillovers of natural conditions from neighboring regions. Ignoring this spatial dependency can lead to biased estimates of DT's effects. Hence, introducing spatial econometric models to examine both the direct and spatial spillover effects of DT on food security is necessary to reveal their regional linkage effects.

In view of this, this paper aims to construct an integrated analytical framework through which DT empowers food security. Within this framework, we analyze the issue from the perspectives of climate, land, and the division of labor. The main contributions of this article are threefold. First, at the theoretical level, we construct a dynamic theoretical model that incorporates technology adoption decisions, risk aversion, and factor market equilibrium (including land and services). This model mathematically and rigorously derives the mechanisms by which DT affects food production through three pathways: optimizing agricultural socialized services, facilitating land transfer, and mitigating climate risks. It thus provides a solid microfoundation for subsequent empirical research. Second, at the empirical level, using panel data from 285 prefecture-level cities in China from 2011 to 2024, we innovatively construct a city-level comprehensive DT development index. This index combines artificial intelligence patent data (representing cutting-edge technological innovation) with traditional digital indicators (representing infrastructure penetration), thereby offering a more comprehensive representation of regional DT levels. Furthermore, this study employs a dynamic spatial Durbin model (SDM) to systematically examine the direct and spatial spillover effects of DT on food security, and uses a mediation model to empirically test the theoretical mechanisms. Third, at the level of policy implications, through in-depth heterogeneity analysis, this paper identifies differences in the empowering effects of DT across various contexts: different regional classifications (major grain-producing areas vs. non-major producing areas), different natural endowments (terrain undulation), and different economic environments (levels of financial development). These findings provide precise decision-making references for governments to formulate digital village strategies and smart agriculture support policies that are tailored to local conditions.

## 2 Literature review

### 2.1 Digital technology and agricultural development

As a deepening and integration of information technology, DT is fundamentally reshaping traditional agriculture. The academic community has engaged in extensive and in-depth discussions on the impact of DT on agricultural development, yielding a rich body of research. These studies can be broadly summarized into three levels.

The first level is the micro-subject level, which primarily focuses on the impact of DT on farmers' production and operation behaviors. Research generally suggests that the application of DT can significantly improve farmers' access to information and reduce information asymmetry (19, 20). For example, through mobile Internet access, farmers can obtain real-time information on market prices, weather warnings, and pest control, thereby optimizing planting decisions and reducing production blindness. The development of digital inclusive finance has effectively alleviated credit constraints faced by smallholders, enhanced their willingness and ability to adopt new crop varieties and technologies, and consequently increased agricultural productivity (21, 22). In addition, the rise of e-commerce platforms has opened new channels for agricultural product sales, alleviated market access constraints, increased farmers' operating income, and given rise to new entrepreneurial models such as live-streaming sales (23). However, studies have also pointed out the existence of a “digital divide” in the application of DT. Farmers with lower educational attainment and those who are older may lack the digital skills necessary to fully benefit from the digital dividend, which could potentially exacerbate inequality across groups (24, 25).

The second level is the meso-industrial level, which examines how DT drive the transformation and upgrading of the agricultural industrial chain. DT have enabled precise monitoring and intelligent management of agricultural production processes through devices such as the Internet of Things and sensors, thereby fostering the emergence of smart agriculture and precision agriculture (26–29). This development not only reduces the consumption of production inputs such as water, fertilizers, and pesticides, achieving cost reduction, efficiency improvement, and green production, but also optimizes crop distribution and resource allocation through big data analytics. In the downstream segments of the supply chain, technologies such as blockchain have been applied to establish traceability systems for agricultural products, thereby strengthening food safety and consumer trust. At the same time, DT have facilitated the deep integration of agriculture with secondary and tertiary industries. For example, the development of rural tourism and contract farming has extended the agricultural industrial chain and enhanced the value chain. Some studies have further found that the growth of the digital economy can improve overall regional economic performance through the effects of technological innovation and industrial structure upgrading, and agriculture, as an integral component of this process, has also benefited (30).

The third dimension concerns the macro-environment and sustainable development. DT show considerable potential in addressing climate change and fostering sustainable agricultural development. For instance, remote sensing technology and meteorological big data can provide precise support for agricultural climate risk assessment and early warning, enabling agricultural producers to adopt adaptive measures proactively (31). DT can also contribute indirectly to carbon reduction in the agricultural sector by optimizing the energy mix and enhancing energy efficiency (32, 33). Moreover, a green financial system empowered by DT can channel funds more effectively toward environmentally sustainable agricultural projects, thereby accelerating the green transformation of agricultural production methods (34, 35). International studies further underscore that Digital Agriculture Services play a pivotal role in enhancing smallholder productivity in low-income countries and in addressing climate change (36–38).

#### 2.1.1 Factors influencing food security

Food security is a multidimensional and complex concept influenced by a wide array of factors. Conventional research has predominantly focused on natural endowments, technological advancements, policy support, and market environments (39). Natural endowments, such as the quantity and quality of arable land resources, the abundance of water resources, and climatic conditions, are fundamental determinants of a country's or region's food production potential (40). Technological advancements, particularly green revolution technologies including seed breeding, fertilizer and pesticide application, and mechanization, have served as the primary impetus behind the substantial growth in global food output over recent decades. Policy support, encompassing agricultural subsidies, price support mechanisms, food reserves, and investment in research and development, plays a critical role in stabilizing farmers' income expectations, stimulating food production, and smoothing market fluctuations (41). The market environment involves the efficiency of the land transfer market, the agricultural inputs market, the labor market, and the agricultural product trading system (42). Collectively, these factors shape the allocative efficiency of agricultural production factors and the accessibility of food.

As the global environment undergoes continuous change, the impact of emerging risk factors on food security has become increasingly prominent. Climate change is widely recognized as one of the most severe long-term threats to global food security in the 21st century. Rising temperatures, shifting precipitation patterns, and the increasing frequency of extreme weather events such as droughts and floods directly impair crop growth, development, and yield stability, posing a particularly serious threat to vulnerable agricultural ecosystems (43, 44). Furthermore, in an increasingly globalized context, external shocks including international market price fluctuations, trade frictions, and geopolitical conflicts may also threaten domestic food security by disrupting food imports and exports and undermining supply chain stability (45–47).

#### 2.1.2 The connection between DT and food security

Although the literature on both digital technologies and food security provides a solid foundation for understanding each domain separately, few studies have directly linked the two for systematic investigation. Existing explorations have primarily identified preliminary trends and gaps that require further examination.

A growing body of research has begun to focus on the potential of DT in ensuring food security. For instance, scholars have argued that Industry 4.0 technologies such as the Internet of Things, artificial intelligence, and big data analytics can substantially increase food production and resource use efficiency by enabling precision agriculture and smart farming, and represent important pathways toward achieving the United Nations Sustainable Development Goal of Zero Hunger (64, 65). Several review articles have systematically examined the current state of digital agricultural technology applications in developing countries, contending that digital advisory services and market information systems positively affect the productivity and income of smallholder farmers, thereby indirectly enhancing their food security (48). However, the majority of these studies are qualitative analyses or case studies that lack rigorous econometric tests based on large-scale data, which limits their ability to establish universal causal effects. A limited number of empirical studies have attempted to quantify the impact of DT on agricultural output. For example, research employing provincial panel data has found that the development of digital inclusive finance exerts a significant positive effect on food production (49, 63). These studies, however, tend to equate food production with food security. Yet food security encompasses not only output but also the stability, accessibility, and sustainability of that output. Furthermore, these studies often adopt a relatively narrow measurement of DT and do not sufficiently explore the underlying mechanisms.

#### 2.1.3 Research gaps

Overall, the existing literature provides an important theoretical basis and empirical reference for this study, but there is also obvious room for expansion. First, there is insufficient exploration of the “black box” problem of DT empowering food security in existing studies, and there is a lack of an integrated theoretical framework to explain its internal transmission path. This paper attempts to open the “black box” by constructing a theoretical model to incorporate three key elements—agricultural socialized services, land transfer, and climate risk—into the analytical framework. Second, the measurement methods of DT in existing research are not being improved and do not fully reflect the innovative core of the new generation of information technology. This paper aims to capture the essence of DT development more accurately by constructing a composite index that includes artificial intelligence patents. Third, existing research generally neglects the spatial dimension of DT and food security and fails to examine its spatial spillover effects. This paper introduces a spatial econometric model, aiming to make up for this deficiency and provide new evidence for understanding regional synergistic development.

## 3 Theoretical mechanisms and research hypotheses

In order to systematically explain the inherent logic of DT empowering food security, this section constructs a theoretical model that includes agricultural producers (farmers), agricultural social service organizations, and land markets. The model aims to reveal how DT affects regional food output by influencing information access, transaction costs and risk perception, and then the adoption of agricultural socialized services, land transfer decisions and adaptation to climate risks.

### 3.1 Theoretical models

Consider a representative agricultural producer (farmer) whose aim is to maximize expected utility. The production function of a farmer depends on the land, labor, capital it invests and the agricultural technology it adopts. To simplify the analysis, we divide the farmers' decision-making process into two stages. In the first stage, farmers decide whether to transfer out part or all of their land and whether to purchase socialized services from the service market. In the second stage, farmers use their own or leased land and purchased services for production and face output uncertainty brought about by climate risks.

The utility function of farmers is in the form of constant relative risk aversion (CRRA) $\left[U\left(W\right)=\frac{W^{1-\gamma }}{1-\gamma }\right]$, where *W* is the final wealth, and [[Inline Image]] is the risk aversion coefficient. The farmers' grain output function is set as:


$$\begin{array}{l} Y=A\cdot f\left(L,K,S\right)\cdot \theta \end{array}$$ (1)

In Equation 1, *Y* is grain output, *A* is technical level, *f*(·) is a production function with constant returns to scale, *L* is land input, *K* is input of other factors such as capital and labor, and *S* is the level of agricultural socialized services adopted. The key point is θ. It is a random variable that represents the climate shock and follows a probability distribution with a mean of 1, and a variance of $\sigma_{\theta }^{2}$. θ>1 represents favorable climate conditions, and θ>1 is unfavorable climate shocks (such as droughts and floods). DT *D* plays multiple roles in this model. First of all, DT can reduce the uncertainty of climate risks through big data analysis and precise predictions. We assume that the application of DT can reduce the perceived variance of climate risk from $\sigma_{\theta }^{2}$ to $\sigma_{\theta }^{2}\left(D\right)$, where $\frac{\partial \sigma_{\theta }^{2}\left(D\right)}{\partial D}<0$. Secondly, DT reduces information asymmetry and transaction costs, influencing the efficiency of the land transfer market and the socialized service market.

### 3.2 DT, agricultural socialized services and food security

Agricultural socialized services, such as unified plowing, sowing, management, harvesting and drying, can overcome the shortcomings of small-scale farmers in terms of technology, capital and machinery, achieve economies of scale and technology diffusion, and are the key to improving agricultural production efficiency. However, the operation of the service market faces a serious problem of information asymmetry: farmers have difficulty accurately assessing the quality of services, and service providers have difficulty precisely matching farmers' demands.

DT has greatly reduced this information asymmetry by building information platforms and implementing online monitoring and evaluation feedback mechanisms for the service process. Let the cost of adopting socialized service *S* be *C*(*S*), and the expected output gain it brings be *E*[Δ*Y*(*S*)]. In the absence of DT, due to the lack of transparency in information, farmers' perceived quality of service is uncertain, resulting in a lower assessment of the expected output gain or a higher perception of the risk of adopting the service. The introduction of DT has raised farmers' expectations of service outcomes by providing more accurate service information and quality assurance, that is $\frac{\partial E\left[\Delta Y\left(S\right)\right]}{\partial D}>0$, and has reduced the perceived risk of adopting services.

As a result, at a given cost, the higher the level of DT in a region, the more likely farmers are to adopt a higher level of socialized services. That is, the optimal service adoption level *S* is an increasing function of the DT level *D*, and *S* = *S*(*D*), at same time, $\frac{dS^{*}}{dD}>0$. Since socialized services are a positive input $\frac{\partial f}{\partial S}>0$ in the production function, a higher level of service adoption will directly lead to an increase in food output. Based on the above analysis, the first research hypothesis is proposed:

*Hypothesis 1 (H1): DT has a positive impact on food security by promoting the improvement of agricultural socialized service levels*.

### 3.3 DT, Land transfer and food security

Fragmented land management is a major obstacle to the scale, mechanization and modernization of China's agriculture. Land transfer is the fundamental way to achieve moderate-scale land management and improve the efficiency of land allocation. However, the land transfer market also has high transaction costs, including information search costs, negotiation costs and contract execution supervision costs.

DT, especially land transfer information platforms based on geographic information systems (GIS) and the Internet, can effectively gather and publish information on land supply and demand, significantly reducing the cost of information search. Technologies such as smart contracts can regulate transaction processes and reduce negotiation and signing costs. This makes potential deals that would otherwise be unachievable due to high transaction costs possible, thereby increasing the activity and efficiency of the land transfer market.

Let *T* represent the efficiency of the land transfer market, which is inversely proportional to transaction costs. The development of DT has reduced transaction costs, and therefore $\frac{\partial T}{\partial D}>0$.

Higher market efficiency means that land can flow more smoothly from less productive farmers to more productive new types of business entities, such as family farms and cooperatives, to achieve optimal allocation and large-scale operation of land resources. Scale operations not only make better use of large agricultural machinery, but also share the fixed costs of adopting new technologies, thereby increasing output per unit area and total output. As a result, DT promotes large-scale agricultural operations by enhancing the efficiency of land transfer, thereby increasing the total volume and efficiency of food production. Based on the above analysis, the second research hypothesis is proposed:

*Hypothesis 2 (H2): DT has a positive impact on food security by accelerating land transfer and promoting large-scale agricultural operations*.

### 3.4 DT, climate risks and food security

Extreme weather events brought about by climate change are a major source of uncertainty for food production. This risk not only directly results in production losses, but also further suppresses output by influencing farmers' production decisions. Risk-averse farmers tend to adopt conservative production strategies in the face of high uncertainty, such as reducing high-risk, high-return productive inputs (like high-quality but less resilient seeds, fertilizers), which leads to a decline in expected output.

DT enhance the resilience of agricultural systems to climate risks in multiple ways. First, sensors based on the Internet of Things (IoT) can monitor soil moisture, crop growth and meteorological data in real time. Combined with big data analysis and artificial intelligence models, early warnings of disasters such as droughts and pests and diseases can be achieved. This precise risk warning information (the $\sigma_{\theta }^{2}\left(D\right)$ decline in our model) reduces the production uncertainty perceived by farmers. Secondly, smart agriculture measures such as precision irrigation and variable fertilization supported by DT can dynamically adjust resource input in response to real-time environmental changes, enabling crops to maintain a relatively stable growth state even under adverse climatic conditions, that is, enhancing the robustness of the output function to climate shocks θ. When the perceived risk $\sigma_{\theta }^{2}\left(D\right)$ decreases, risk-averse farmers will be more willing to increase productive input *K*, thereby raising expected output *E*[*Y*]. In addition, greater resilience means less actual yield loss when facing the same climate shock. These two aspects work together not only to increase the average yield of food, but more importantly to enhance the stability of production, which is one of the core essentials of food security. Based on the above analysis, a third research hypothesis is proposed:

*Hypothesis 3 (H3): DT has a positive impact on food security by enhancing early warning and resilience against climate risks, stabilizing and increasing food production*.

### 3.5 Spatial spillover effects of DT

Spatial economics and technology diffusion theory posit that economic activities across regions are not isolated; rather, mutual influence and interdependence generally prevail, giving rise to spatial spillover effects (50). As a general-purpose technology characterized by high permeability, strong universality, and network externalities, the impact of DT on food security is bound to transcend administrative boundaries and generate significant spatial spillovers (51). This positive spatial spillover effect is realized primarily through the following interrelated channels.

From the perspective of knowledge spillover and technology diffusion theory, DT inherently possess the characteristics of low marginal cost of replication and cross-border transmissibility, which constitute the technical basis for spatial spillovers. Breakthroughs in DT innovation within a region (for example, the development of intelligent irrigation decision-making systems and AI-based pest and disease identification models) and their applications (such as the construction of regional agricultural big data platforms) essentially represent new forms of knowledge. Owing to information and communication technologies, the costs of codifying and disseminating such knowledge are extremely low, enabling it to rapidly cross geographical barriers and be acquired, learned, and re-developed by agricultural operators in adjacent and even more distant regions (52). This intangible flow of knowledge can effectively enhance agricultural technical efficiency and management practices in surrounding areas, thereby contributing to improvements in their food security.

From the perspective of the imitation and learning mechanisms in new institutional economics, successful DT practices in agriculture generate a strong demonstration effect (53). When digital agricultural models in a given region, such as agricultural e-commerce and remote sensing based precision fertilization, achieve significant yield increases, income growth, or disaster reduction effects, signals of success are rapidly transmitted to surrounding areas through channels including the agricultural industrial chain, professional farmer cooperatives, and policy learning among local governments. Driven by both risk aversion and the pursuit of benefits, farmers, enterprises, and governments in neighboring areas tend to imitate and learn from these established experiences and technologies, thereby reducing the costs and uncertainties of local experimentation and forming a virtuous cycle of “the first to drive the second to the third” (54). This represents an important manifestation of the spatial spillover effect at the behavioral level.

From the perspective of new economic geography and factor mobility theory, DT have facilitated the cross-regional mobility and integration of agricultural production factors by optimizing resource allocation (60). Digital platforms can substantially reduce the search and matching costs for both the supply and demand sides of factor markets, thereby accelerating the spatial reallocation of factors. For instance, cross-regional agricultural machinery services enabled by DT allow agricultural machinery from one region to flow in an orderly manner to neighboring areas through online scheduling platforms during peak farming seasons, directly exporting mechanization capacity. Similarly, remote diagnosis systems enable agricultural technical experts in one area to provide real-time guidance to farmers in surrounding areas, thereby realizing the indirect flow of human capital. In addition, DT intensify inter-regional market competition and linkages by reshaping the boundaries of agricultural product markets. When a region reduces production costs or enhances product quality through DT, it generates competitive pressure on agricultural markets in neighboring regions, compelling them to innovate and improve efficiency, thereby strengthening the food security capacity of the entire region at a macro level.

In summary, the impact of DT on food security is not confined within the region. The development level of DT in a region not only directly affects the local agricultural production function to ensure local food security, but also positively promotes the total factor productivity, production stability and market accessibility of food in neighboring regions through the intangible diffusion of knowledge, the demonstration of successful practice, and the optimal allocation of enabling production factors across regions. Thus, a positive spatial spillover effect is formed. Based on the above analysis, the fourth research hypothesis is proposed.

*Hypothesis 4 (H4): The impact of DT on food security has a positive spatial spillover effect, that is, the development of DT in this region can promote the improvement of food security levels in neighboring regions*.

## 4 Research design

### 4.1 Model setting

To test the spatial spillover effect of DT on food security, this paper constructs a spatial econometric model. Considering that the level of food security may be affected by the previous level, showing a certain path dependence, and the impact of DT may also have a time lag, this paper introduces the first-order lag term of the explained variable in the model to construct a dynamic spatial panel model. The Spatial Durbin model (SDM), which takes into account both the explained variable and the spatial lag term of the explanatory variable, is a more common and robust choice. Therefore, this paper sets up the following dynamic spatial Durbin model:


$$\begin{array}{l} FoodSec_{it}=\;\alpha_{0}+\rho WFoodSec_{it}+\tau FoodSec_{i,t-1}+\eta WFoodSec_{i,t-1}\\ \;+\beta_{1}Digital_{it}+\theta_{1}WDigital_{it}+\overset{K}{\underset{k=1}{\sum }}\beta_{k}X_{kit}+\overset{K}{\underset{k=1}{\sum }}\theta_{k}WX_{kit}\\ \;+\mu_{i}+\lambda_{t}+\varepsilon_{it} \end{array}$$ (2)

In Equation 2, the subscripts i and t represent the city and the year respectively. *FoodSec*_*it*_ is the explained variable, that is, the food security level of city *i* in year *t*. *Digital*_*it*_ is the core explanatory variable, that is, the level of DT development. *X*_*it*_ is a series of control variables. ρ is the spatial lag coefficient of the explained variable. It measured the impact of food security levels in neighboring regions on the region. τ is the time lag coefficient of the explained variable, measuring the impact of the previous period's food security level on the current period. η is the coefficient of the time and space lag term. β_1_ is the coefficient of the direct impact of DT on food security. θ_1_ is the spatial lag coefficient of DT, measuring the impact of DT development in neighboring regions on food security in that region. β_*k*_ and θ_*k*_ are the coefficients of the control variable and its spatial lag term respectively. *W* is the spatial weight matrix. This paper uses a spatial weight matrix based on the reciprocal of geographical distance, that is, if the geographical distance between cities *i* and *j* is *d*_*ij*_, then the weight matrix elements *w*_*ij*_ = 1/*d*_*ij*_, and the diagonal elements *w*_*ij*_ = 0. μ_*i*_ and λ_*t*_ respectively represent the urban fixed effect and the time fixed effect, and [[Inline Image]]is the random perturbation terms. To test the mediating mechanism, this paper uses a three-stage mediating effect model for analysis. This paper constructs the mediating effect model as follows:


$$\begin{array}{l} FoodSec_{it}=cDigital_{it}+\sum \gamma_{k}X_{kit}+\mu_{i}+\lambda_{t}+\varepsilon_{it} \end{array}$$ (3)

$$\begin{array}{l} FoodSec_{it}=cDigital_{it}+\sum \gamma_{k}X_{kit}+\mu_{i}+\lambda_{t}+\varepsilon_{it} \end{array}$$ (4)

$$\begin{array}{l} M_{it}=aDigital_{it}+\sum \delta_{k}X_{kit}+\mu_{i}+\lambda_{t}+\varepsilon_{it} \end{array}$$ (5)

$$\begin{array}{l} FoodSec_{it}=c^{\prime }Digital_{it}+bM_{it}+\sum \zeta_{k}X_{kit}+\mu_{i}+\lambda_{t}+\varepsilon_{it} \end{array}$$ (6)

Equations 3 to 6, *M*_*it*_ represent mediating variables, namely agricultural socialized service level, land transfer efficiency, and climate risk resilience (CRR). Coefficient c represents the total effect of DT on food security, coefficient *a* represents the effect of DT on the mediating variable, and coefficient *b* represents the effect of the mediating variable on food security after controlling for the impact of DT. The size of the mediating effect is *a*×*b*.

### 4.2 Variable definitions

#### 4.2.1 Explained variable

Food security level (FoodSec). The core of food security is ensuring an adequate and stable supply of food. This paper draws on existing research and uses per capita grain production (tons per person) as the core proxy variable for measuring the level of food security in a region. This indicator visually reflects the region's food supply capacity relative to its population size. The data is derived from the China Urban Statistical Yearbook and provincial and municipal statistical yearbooks, calculated by dividing the total grain output of each prefecture-level city by the permanent resident population. While per capita grain production is the most commonly used indicator for food security at the city level in China, we recognize that food security is multidimensional. In our main analysis, we focus on the availability dimension.

#### 4.2.2 Core explanatory variables

Level of DT development (Digital). To fully depict the development of DT, this paper constructs a composite index. Existing research mostly uses a single indicator or focuses on digital finance, failing to fully reflect the cutting-edge dynamics of technological innovation. This paper argues that the development of DT encompasses two dimensions: “technological innovation” and “facility accessibility”. Therefore, the composite index of this paper consists of two parts: (1) the number of artificial intelligence patents. Artificial intelligence is a strategic technology leading a new round of technological revolution, and the number of its patents can precisely reflect a region's innovation capacity and potential in the field of cutting-edge DT (11, 55). This paper uses the “China Prefecture-level City Artificial Intelligence Patent Dataset”, which is based on patent data from the National Intellectual Property Administration and is matched to the prefecture-level city level in accordance with the “Patent Classification System for Key Digital Technologies (2023)” to screen out AI-related patents. (2) Traditional digital infrastructure. This part reflects the extent to which DT is applied and the underlying conditions. This paper selects two classic indicators: the number of Internet broadband access users and the number of mobile phone users, and calculates their penetration rate relative to the total population. (3) This paper uses the entropy method to combine the three standardized indicators of the number of AI patents, the Internet penetration rate, and the mobile phone penetration rate into a comprehensive index of DT for a city.

#### 4.2.3 Mediating variables

Agricultural socialized service (ASS). The core of agricultural socialized services lies in enhancing agricultural production efficiency through specialized services. This paper, drawing on relevant research, uses the level of agricultural mechanization as its proxy variable, specifically measured by the ratio of the total power of agricultural machinery (ten thousand kilowatts) to the total sown area of crops (thousand hectares). Mechanized services are one of the most important and standardized forms of agricultural socialized services and can well reflect the extent of service coverage.

Land transfer efficiency (LTE). In our main analysis, we employ the number of legal entities in agriculture, forestry, animal husbandry, and fisheries as a proxy for land transfer efficiency. Legal entities, such as agricultural enterprises and large cooperatives, are typically the primary actors that acquire land through transfers and engage in large scale operations. An increase in their number, to some extent, reflects the intensity of land transfer activities and the advancement of scaled operations. We acknowledge that this indicator is indirect and may not fully capture the actual area transferred or the quality of transfer contracts.

Climate risk resilience (CRR). DT can help agriculture withstand climate risks, and the effect should be reflected in reducing the impact of climate fluctuations on yields. This paper constructs an indicator to measure the impact of climate factors on food production. The specific approach is: First, calculate the absolute value of the deviation rate of the annual grain yield per city (total grain output/sown area) relative to its moving average of the previous 5 years, which reflects the interannual fluctuations in production. Secondly, considering that climate risk mainly stems from abnormal precipitation and temperature, we use the degree of deviation of current year's precipitation and average annual temperature from historical averages to represent climate shock. Finally, we divide the volatility of grain yield per unit area by the intensity of climate shock to get a standardized climate risk impact coefficient. For the sake of explanation, this paper takes the opposite of the indicator, denoted as CRR, the larger the value, the stronger the resilience to climate risk. The meteorological data is from China Meteorological Data Network.

#### 4.2.4 Control variables

To mitigate the bias of omitted variables, this paper controls for the following city-level variables that may affect food security: Economic development level (LED) : measured as the logarithm of real GDP per capita. Regions with higher levels of economic development typically have stronger financial resources to support agricultural development. Industrial structure (Ind_str): measured by the proportion of the added value of the secondary industry to GDP. Industrial structure affects the allocation of labor and capital between urban and rural areas and among different industries. Government intervention (Gov) : measured by the proportion of local government spending on agriculture, forestry and water affairs to general budget spending. Urbanization level: measured by the proportion of Urban population to total population. Urbanization affects the supply of rural labor and the demand for commercial grain. Finance: measured by the combined balance of deposits and loans of financial institutions at the end of the year as a proportion of GDP. Financial development affects the availability of agricultural investment. Human capital (Edu) : measured by the proportion of college students to the total population. High levels of human capital facilitate the adoption and diffusion of new technologies. Transportation infrastructure (Road) : measured by per capita urban road area, reflects the convenience of transporting agricultural supplies and the circulation of agricultural products.

### 4.3 Data sources

The sample of this study is panel data from 285 prefecture-level and above cities in China from 2011 to 2024. The data for this study were sourced from an open database. The main sources are as follows: China Economic and Social Big Data Research Platform (https://data.cnki.net/), economy prediction system (EPS) database (https://www.epsnet.com.cn/index.html#/index), and various provinces and cities. Some economic variables were logarithmed to eliminate heteroscedasticity. All nominal variables were deflated with 2011 as the base period. Descriptive statistics of the primary variables are shown in Table 1.

**Table 1:** Descriptive statistics.

Variable	Symbols	Observations	Mean	Standard deviation	Minimum value	Maximum
Food security	FoodSec	3,990	0.583	0.612	0.001	4.891
Digital technology	Digital	3,990	0.157	0.129	0.011	0.987
Agricultural socialized services	ASS	3,990	5.872	3.104	0.453	18.921
Land transfer efficiency	LTE	3,990	156.4	123.8	12	897
Climate risk resilience	CRR	3,990	−0.215	0.184	−0.982	−0.005
Level of economic development	LED	3„990	10.85	0.631	8.987	12.54
Industrial structure	Ind_str	3,990	45.12	12.34	10.21	78.95
Government intervention	Gov	3,990	2.876	1.543	0.321	9.874
Urbanization rate	Urban	3,990	58.91	15.67	25.43	98.76
Financial development	Finance	3,990	2.154	0.876	0.765	5.873
Human capital	Edu	3,990	3.124	2.543	0.543	15.87
Transportation infrastructure	Road	3,990	18.76	8.987	3.456	56.78

## 5 Empirical results and analysis

### 5.1 Benchmark regression results

Before the estimation of the spatial econometric model, this paper first determines the most appropriate model form through the Lagrange multiplier (LM) test, Hausman test, and likelihood ratio (LR) test. The results of the LM test significantly rejected the null hypothesis of no spatial autocorrelation at the 1% level, indicating the necessity of using a spatial econometric model. The Hausman test results support the use of a fixed-effect model. Both the LR test and the Wald test results significantly rejected the assumption that SDM models could be simplified to spatial autoregressive (SAR) model or spatial error model (SEM) at the 1% level. Therefore, this paper ultimately chose to use the dynamic spatial Durbin model with bidirectional fixed effects in space-time for estimation. Due to the endogeneity associated with the lag term and the perturbation term in the dynamic panel model, this paper uses Arellano-Bond's differential generalized method of moments (GMM) for estimation. Table 2 reports the benchmark regression results.

**Table 2:** Benchmark regression results.

Variables	OLS	FE	SAR	Dynamic SDM
	(1)	(2)	(3)	(4)
	FoodSec	FoodSec	FoodSec	FoodSec
Digital	0.285^***^ (0.041)	0.198^***^ (0.053)	0.175^***^ (0.049)	0.153^***^ (0.042)
L.FoodSec				0.211^***^ (0.035)
*W* ^*^ Digital				0.089^**^ (0.040)
ρ (*W* ^*^ FoodSec)			0.254^***^ (0.061)	0.231^***^ (0.058)
η (*W* ^*^ l. fodsec)				0.067^**^ (0.038)
Controls	Yes	Yes	Yes	Yes
City FE	No	Yes	Yes	Yes
Year FE	Yes	Yes	Yes	Yes
Observations	3,420	3,420	3,420	3,135
AR(1) *p*-value				0.008
AR(2) *p*-value				0.254
Hansen *p*-value				0.312

Robust standard errors are in parentheses. ^***^, ^**^, and ^*^ indicate significance at the 1%, 5%, and 10% statistical levels, respectively.

The regression results in Table 2 show that from the mixed OLS model in column (1) to the dynamic spatial Dubin model in column (4), the coefficient of the core explanatory variable Digital is always positive and significant at the 1% level. This initially suggests that the development of DT can indeed significantly enhance food security levels in the region. Column (4) is the result we are most concerned about, with a Digital coefficient of 0.153, indicating that for every unit increase in the local DT composite index after controlling for other factors, the local per capita food production will increase by 0.153 tons directly. The enabling effect of DT on food security runs through the whole chain of “pre-production, in-production and post-production” of agricultural production, whose core is to reconstruct the factor allocation mode, production function form and industrial organization mode of traditional agriculture through the embedding of data, a new production factor.

(1) At the pre-production stage of agriculture, DT enhance the precision of factor supply and drive the intelligent transformation of the agricultural innovation paradigm. This stage constitutes the factor preparation period for grain production, with the core task of ensuring the supply of high-quality seeds, cultivated land resources, and institutional arrangements. In seed industry innovation, DT are propelling the breeding paradigm from “trial and error” toward “intelligent design.” On the one hand, intelligent breeding technologies based on genomics and big data analytics can substantially shorten the breeding cycle and enable the targeted selection of desirable traits such as stress resistance and high yield, thereby strengthening the core competitiveness of the seed industry. On the other hand, the engagement of e-commerce platforms and social media has reshaped the traditional promotion model of the seed sector, compelling seed enterprises to shift from pure production and sales toward the integration of R&D, service, and marketing through data interaction and brand management between supply and demand (58). At the level of cultivated land factor management, digital supervision platforms built upon high-resolution remote sensing, video surveillance, and geographic information systems realize the dynamic monitoring of cultivated land quality, planting structure, and utilization intensity. These platforms provide precise data support for the implementation of agricultural functional zoning and crop rotation and fallow systems, thereby optimizing the spatial layout of grain production (45).
(2) At the agricultural production stage, DT promote the automation of production processes and the intelligent management of the environment. This stage constitutes the direct formation period of grain output, and the core contribution of DT lies in resolving the dilemma of experiential dependence and resource misallocation inherent in traditional agriculture. First, the diffusion of smart equipment is reshaping agricultural production. Leveraging Beidou navigation and IoT technologies, unmanned agricultural machinery and 5G-enabled plant protection drones have achieved precision in sowing, fertilization, and pesticide application, significantly enhancing factor utilization efficiency and operational accuracy. Second, the digital transformation of facility agriculture has given rise to a new production paradigm. Smart greenhouses integrate sensors and AI algorithms to realize closed-loop control of environmental factors such as light, temperature, water, gas, and fertilizer, thereby creating an optimal micro-environment for crop growth and improving the marginal returns on resource inputs (62). Third, DT have strengthened the risk response capacity of agriculture. Through the integration of meteorological big data with pest and disease early warning models, an intelligent disaster prevention and mitigation system can be established, effectively mitigating the impact of natural risks on grain yield.
(3) At the post-harvest stage, DT promote the visualization of the agricultural circulation system and the intelligentization of inventory management. This stage is critical to realizing food value and controlling losses. DT have reconstructed the food supply chain and value chain by dismantling information barriers. In the harvesting process, intelligent harvesters equipped with remote sensing and yield measurement systems enable real-time collection and plot-level attribution of yield data, thereby providing a closed data loop for the next round of precision planting. In the circulation process, the proliferation of digital trading platforms and mobile applications has shortened the distance between farmers and markets, mitigated supply-demand mismatches arising from information asymmetry through real-time monitoring of procurement and sales data and the transparency of price signals, and enhanced the operational efficiency of grain markets (56). In the storage process, smart grain warehouses, relying on temperature and humidity sensors, gas sensors, and intelligent ventilation systems, achieve real-time perception and automatic control of grain storage conditions, curbing mildew and losses at the source and ensuring grain quality and safety throughout the storage cycle.

The estimates of the spatial lag terms provide additional important information. The spatial autoregressive coefficient of the dependent variable is significantly positive at the 1% level (0.231), indicating a significant positive spatial correlation in food security across regions; an improvement in food security in one region can generate positive demonstration and driving effects on surrounding areas. The coefficient of the spatial lag of the core explanatory variable (*W* ^*^ Digital) is 0.089 and significant at the 5% level, suggesting that the development of DT in neighboring regions also exerts a significant promoting effect on local food security, thereby providing preliminary support for hypothesis H4. The coefficient of the time-lagged dependent variable (L.Fodsec) is significantly positive, indicating that food security exhibits a dynamic inertial characteristic. The diagnostic test results for the dynamic SDM model reveal that the AR (1) statistic is significant whereas the AR (2) statistic is not, confirming the absence of second-order autocorrelation in the differenced residuals. The Hansen test *p*-value exceeds 0.1, indicating that the instruments are valid.

Since the point estimation results cannot be directly interpreted as marginal effects, we further perform partial differential decomposition on the estimation results of the dynamic SDM model to obtain the direct effects, indirect effects (spatial spillover effects), and total effects of DT on food security. The results of the spatial effect decomposition are shown in Table 3.

**Table 3:** Results of spatial effects decomposition.

Effect	Short-run	Long-run
Direct Effect	0.158^**^ (0.045)	0.235^***^ (0.061)
Indirect Effect (Spillover)	0.102^**^ (0.048)	0.151^**^ (0.069)
Total Effect	0.260^***^ (0.066)	0.386^***^ (0.092)
Controls	Yes	Yes
City FE	Yes	Yes
Year FE	Yes	Yes

Robust standard errors are in parentheses. ^***^ and ^**^ indicate significant at the 1% and 5% levels respectively.

The effect breakdown results in Table 3 clearly reveal the path of the impact of DT on food security. In the short term, the direct effect of DT is 0.158 and the indirect effect is 0.102, both of which are statistically significant. This means that the development of DT in a region can not only directly promote local food security, but also, through spatial spillover, boost the level of food security in surrounding areas. The size of the indirect effect is about 65% of the direct effect, indicating that the spatial spillover effect cannot be ignored. In the long term, given the dynamic adjustment process, the impact of DT will be further amplified, with the long-term direct and indirect effects reaching 0.235 and 0.151 respectively. This fully confirms hypothesis H4, that DT has a significant positive spatial spillover effect on food security.

### 5.2 Robustness test

To ensure the reliability of the benchmark regression results, a series of robustness tests were conducted in this paper.

(1) Replace the core explanatory variable. We replace the DT Composite Index (Digital) with two single-dimensional proxy variables: one is to use only the number of AI patents (AI_patent) to represent cutting-edge technological innovation; The second is to use the Peking University Digital Inclusive Finance Index (DIFI) to represent the inclusive finance dimension of DT. The regression results are shown in columns (1) and (2) of Table 4. It can be seen that regardless of which proxy variable is used, both the direct effect and the spatial spillover effect are significantly positive, consistent with the baseline regression results. These results suggest that the positive role of DT in promoting food security is robust.
(2) Replace the spatial weight matrix. Benchmark regression uses the reciprocal matrix of geographical distances. To test the sensitivity of the results to the weight matrix Settings, we re-estimated using the adjacency matrix (1 if the two cities are geographically adjacent, or 0 if not) and the economic geography nesting matrix (taking into account the gap in geographical distance and economic development levels). The results in columns (3) and (4) of Table 4 show that there is no substantial change in the core conclusion after the weight matrix is changed.
(3) Generalized spatial two-stage least squares (GS2SLS) estimation. Although the difference GMM estimator effectively addresses endogeneity in dynamic panel models, the spatial lag terms of explanatory variables in a spatial panel setting may still be a potential source of endogeneity. To further test the robustness of our main findings against this concern, we employ GS2SLS. This approach instruments the spatial lag terms using higher-order spatial lags of the exogenous variables, thereby providing consistent estimates in the presence of spatial dependence and endogenous regressors. Specifically, we re-estimate our dynamic spatial Durbin model using GS2SLS with the same set of control variables, city and year fixed effects, and the inverse-distance spatial weight matrix. The results are reported in Table 4. As shown in column (5) of Table 4, the direct effect of DT on food security remains positive and significant at the 1% level (0.147), and the spatial spillover effect (*W* × Digital) remains positive and significant at the 5% level (0.083). The spatial autoregressive coefficient and the time lag coefficient are also highly significant and comparable to the baseline estimates. These results confirm that our main conclusions are not driven by potential endogeneity of the spatial lag terms, and further strengthen the robustness of our findings.
(4) During the sample period, China implemented several national and local policies to promote digital agriculture and rural e-commerce. These policies could confound the estimated effect of DT on food security. To isolate the net effect of DT, we constructed a set of policy dummy variables and incorporated them as additional controls in the dynamic spatial Durbin model. Specifically, we included three dummy indicators. The first, Policy_InfoVillage, equals 1 if a city was covered by the “Information Entering Villages” pilot program, which was launched in 2014 and expanded between 2015 and 2017. Data for this indicator were obtained from the Ministry of Agriculture and Rural Affairs. The second, Policy_Ecommerce, equals 1 if a city was selected as a national “Comprehensive Demonstration of E-commerce into Rural Areas” from 2015 onward, with data from the Ministry of Commerce. The third, Policy_DigitalVillage, equals 1 if a city was part of the “Digital Village” pilot starting in 2018, with data from the Cyberspace Administration of China. All three dummy variables were entered simultaneously into the model. As shown in column (7) of Table 4, after controlling for these policy shocks, the direct effect of DT is 0.144 (*p* < 0.01) and the indirect effect is 0.087 (*p* < 0.05). Both estimates remain statistically significant and close to the baseline results, indicating that our main findings are not driven by these observable policy interventions.
(5) Alternative measure of food security: a composite index. To test whether our results are sensitive to the measurement of food security, we constructed a composite food security index (FoodSec_Comp) using the entropy method. This index integrates four dimensions: Availability: per capita grain output (tons/person); Stability: the inverse of the coefficient of variation of grain yield per unit area over the preceding 5 years; Access: per capita disposable income of rural residents (yuan/person, deflated); Sustainability: grain output per unit of agricultural water consumption (tons/cubic meter). Data for rural income and agricultural water use were obtained from provincial and municipal statistical yearbooks. We then re-estimated the dynamic spatial Durbin model with FoodSec_Comp as the dependent variable. As shown in column (7) of Table 4, the direct effect of DT is 0.124 (*p* < 0.01) and the indirect effect is 0.078 (*p* < 0.05), both remaining positive and significant. The magnitudes are slightly smaller than the baseline, but the sign and significance are consistent. This confirms that our findings are not driven by a single-dimensional measure of food security.

**Table 4:** Results of the robustness test.

Variables	AI_patent	DIFI	Adjacency W	Economic W	GS2SLS	Control policy variables	Composite index
	(1)	(2)	(3)	(4)	(5)	(6)	(7)
Direct Effect	0.085^***^ (0.029)	0.121^***^ (0.038)	0.149 ^***^ (0.048)	0.163^***^ (0.044)	0.147^***^ (0.044)	0.144^***^ (0.023)	0.124^***^ (0.037)
Indirect Effect	0.052^**^ (0.025)	0.076 ^**^ (0.031)	0.095^**^ (0.045)	0.108^**^ (0.051)	0.083^**^ (0.041)	0.087^**^ (0.039)	0.078^**^(0.47)
Controls	Yes	Yes	Yes	Yes	Yes	Yes	Yes
City FE	Yes	Yes	Yes	Yes	Yes	Yes	Yes
Year FE	Yes	Yes	Yes	Yes	Yes	Yes	Yes

^**^ and ^***^ indicate significant at 5% and 1% respectively, and the ones reported in parentheses are robust standard errors.

### 5.3 Endogenous problem

Although benchmark regression takes into account bidirectional fixed effects and the endogeneity of dynamic panels, there may still be issues of missing variables and reverse causality. For example, regions with higher levels of food security may have stronger economic power and willingness to develop DT, which leads to reverse causation. To address this issue, the study introduced instrumental variables (IV) for DT and processed them using the two-stage least squares method (2SLS). Drawing on related studies (57), this paper constructs an interaction term based on the number of historical post offices and global Internet development trends as an instrumental variable for the development of DT. Specifically, we chose the number of post offices in each city in 1984 as the proxy variable for historical information infrastructure and interacted it with the total number of Internet users nationwide in the previous year. The logic of this instrumental variable is that regions with historically better information infrastructure are more likely to be the first to develop DT in the Internet era and thus meet the relevance requirement. However, the distribution of post offices in 1984 was mainly determined by historical factors and was not related to the random disturbance term of the current food security level, satisfying the exogeneity requirement. The number of post offices in 1984 was primarily determined by administrative and geopolitical factors under China's centrally planned system, rather than by local agricultural productivity. Post offices were distributed based on population size and administrative hierarchy, not on agricultural potential. After the reform and opening up, the legacy of historical postal infrastructure influenced early Internet adoption, but the direct impact of 1984 post offices on current grain production has been absorbed by subsequent economic transformations and policy changes. Any remaining historical influence is captured by our inclusion of historical GDP and urbanization rate as additional controls.

Table 5 reports the results of the 2SLS estimation using the instrumental variable. In the first stage of regression, the coefficient of the IV was significantly positive at the 1% level, and the f-statistic was much larger than the empirical threshold of 10, indicating that there was no weak instrumental variable problem. The results of the second-stage regression showed that digital's coefficient remained significantly positive and was numerically larger than that of the baseline regression, indicating that the role of DT in promoting food security was underestimated after taking endogeneity into account. This may be because major grain-producing areas are often economically underdeveloped regions with relatively low levels of DT development, and this negative correlation leads to a downward bias in OLS estimates. In conclusion, the regression results of the instrumental variable method further enhance the reliability of the core conclusions of this paper.

**Table 5:** Results of the endogeneity test.

Variables	First stage	Second stage	First stage	Second stage
	(1)	(2)	(3)	(4)
	Digital	FoodSec	Digital	FoodSec
IV (Post1984 × Internet)	0.078^***^ (0.015)		0.058^***^ (0.021)	
Digital		0.254^***^ (0.078)		0.276^***^ (0.056)
Controls	Yes	Yes		
City FE	Yes	Yes		
Year FE	Yes	Yes		
Historical economic variables	No	No	Yes	Yes
*F*-statistic	27.45		24.87	

^***^Indicates significance at the 1% level. The Kleibergen–Paap rk Wald *F*-statistic exceeds the Stock-Yogo critical value of 16.38 for 10% maximal IV size, rejecting the null of weak instruments.

In order to strengthen the exogeneity requirement of IV as much as possible, when conducting the 2SLS regression analysis, we also added the per capita historical domestic gross domestic product in 1984 and the historical urbanization rate in 1984–1997 as additional control variables. The data of regional GDP and the proportion of urban population in 1984–1997 were obtained from the “Compilation of Chinese Statistical Data from 1949 to 2004” and the statistical yearbooks of each region. These variables eliminated potential interfering factors that might be brought about by the historical path of economic development and urbanization process, thereby strengthening the argument that “endogenous variables” only affect current food security through “direct effects”. The results reported in the revised Table 5 (including the new 2SLS column with historical control variables) show that the coefficient of the direct effect is still positive and significant (0.276, *p* < 0.01), and the F statistic in the first stage is still much higher than 10. Incorporating historical control variables does not significantly change the size or significance of the main effect, which indicates that our initial estimation of endogenous variables was not seriously affected by historical interference factors.

## 6 Mechanism testing and heterogeneity analysis

### 6.1 Mechanism test

The theoretical analysis presented earlier suggests that DT affects food security through three pathways: promoting agricultural socialized services, accelerating land transfer, and enhancing resilience to climate risks. This section uses the mediating effect model to empirically test these three mechanisms. The results are shown in Table 6.

**Table 6:** Results of the mechanism test.

Dependent Var.	(1)	(2)	(3)	(4)	(5)	(6)
	ASS	FoodSec	LTE	FoodSec	CRR	FoodSec
Digital	0.851^***^ (3.55)	0.142^***^ (3.42)	25.34^***^ (3.69)	0.158^***^ (3.67)	0.109^***^ (3.41)	0.161^***^ (3.83)
Service		0.065^***^ (3.61)				
Transfer				0.011^**^ (2.23)		
ClimateRisk						0.287^***^ (3.83)
Controls	Yes	Yes	Yes	Yes	Yes	Yes
City FE	Yes	Yes	Yes	Yes	Yes	Yes
Year FE	Yes	Yes	Yes	Yes	Yes	Yes
Observations	3,135	3,135	3,135	3,135	3,135	3,135
*R*-squared	0.654	0.718	0.589	0.715	0.451	0.723
Sobel Test (*p*-value)	0.000		0.002		0.001	

^***^ *and* ^**^ indicate significance at the 1% and 5% levels respectively, and the *t*-statistic is reported in parentheses.

The results in Table 6 provide strong evidence for the three mechanism hypotheses proposed in this paper. Columns (1) and (2) examine the mediating effect of ASS. Column (1) shows that DT has a significant positive promoting effect on the level of agricultural socialized services (coefficient 0.851). Column (2) When both DT and agricultural socialized services are included in the model, the coefficients for both are significantly positive, and the coefficient for DT (0.142) is lower than the total effect (approximately 0.198 in the baseline regression). The *p*-value of the Sobel test was 0.000, indicating a significant mediating effect. This validates hypothesis H1. DT does guarantee food security by enhancing the level of agricultural socialized services.

Columns (3) and (4) examine the mediating effect of LTE. The coefficient of digital in column (3) is significantly positive, indicating that the development of DT has promoted large-scale agricultural operations represented by the increase of legal entities. In column (4), the coefficients for both digital and LTE are significantly positive. This validates hypothesis H2. DT contributes to food security by accelerating land transfer and scaling up operations. To test the robustness of the mediating effect of land transfer, we replaced the original variable (number of agricultural legal entities) with the number of family farms (log). Family farms are a more direct indicator of land scale operation resulting from land transfers. Due to data availability, this test is conducted for the period 2015–2024. The result shows that, the mediating effect remains positive and significant. The coefficient of DT on food security via family farms is 0.029 (*p* < 0.05), and the Sobel test *p*-value is 0.018. This confirms that our conclusion on the land transfer channel is not sensitive to the choice of proxy variable.

Columns (5) and (6) examine the mediating effect of CRR. Column (5) shows that DT significantly enhances agricultural CRR in regions. In column (6), the coefficients for digital and CRR are also significantly positive. This validates hypothesis H3. DT enhances the stability of food supply by helping agricultural production better cope with climate fluctuations.

In summary, all three mechanisms are empirically supported, with agricultural socialized services being the most important transmission channel for DT to empower food security.

### 6.2 Heterogeneity analysis

The impact of DT on food security may vary depending on regional resource endowments and development stages. This paper conducts heterogeneity analysis from three dimensions: major grain-producing areas, topographic conditions, and financial development levels.

(1) Differences between major grain-producing and non-major grain-producing areas. We divided the samples into two groups for regression: major grain-producing areas and non-major grain-producing areas. The results are shown in columns (1) and (2) of Table 7. Interestingly, the promoting effect of DT on food security is significantly greater in non-major production areas (coefficient 0.189) than in major production areas (coefficient 0.112). This may be because major grain-producing areas have a better agricultural foundation and more mature production models, and the marginal improvement effect brought by DT is relatively small. The introduction of DT can produce a “leapfrog” enabling effect, helping to overcome traditional shortcomings, and thus the effect is more obvious in non-major agricultural areas where the foundation is weak. This reflects the inclusive nature of DT.
(2) Differences in terrain conditions. Areas with flat terrain are more conducive to large-scale mechanized operations and the construction of modern agricultural facilities, and have broader application scenarios for DT. We constructed the terrain complexity index based on the average altitude of the city and the undulation of the terrain, dividing the samples into areas with flat terrain and areas with complex terrain. The results in columns (3) and (4) of Table 7 show that DT contributes significantly more to food security in flat terrain areas (coefficient 0.176) than in complex terrain areas (coefficient 0.095). This suggests that good natural geographical conditions are an important basis for the role of DT.
(3) Differences in the level of financial development. The construction of digital agriculture, whether in terms of hardware investment or software system development, requires substantial financial support. Regions with a higher level of financial development can provide more abundant credit support for the research and application of DT. We divided the sample into high-level and low-level groups based on the median level of financial development. The results in columns (5) and (6) of Table 7 show that the enabling effect of DT is significantly stronger in regions with high levels of financial development (coefficient 0.195) than in regions with low levels of financial development (coefficient 0.103). This indicates that there is a significant complementarity between DT and financial capital, and that coordinated development is necessary to maximize their role in ensuring food security.

**Table 7:** Results of heterogeneity analysis.

Variables	Main production areas	Non-main regions	Flat terrain	Complex terrain	High financial development	Low financial development
	(1)	(2)	(3)	(4)	(5)	(6)
	FoodSec	FoodSec	FoodSec	FoodSec	FoodSec	FoodSec
Digital	0.112^**^ (0.051)	0.189^***^ (0.048)	0.176^***^ (0.045)	0.095^*^ (0.058)	0.195^***^ (0.049)	0.103^*^ (0.053)
Controls	Yes	Yes	Yes	Yes	Yes	Yes
City FE	Yes	Yes	Yes	Yes	Yes	Yes
Year FE	Yes	Yes	Yes	Yes	Yes	Yes

This table reports the direct effects in dynamic SDM models. ^***^, ^**^, and ^*^ represent significance at the 1%, 5%, and 10% statistical levels, respectively. Robust standard errors are reported in parentheses.

## 7 Discussion

This study reveals the profound and multifaceted impact of DT, as a new factor of production, on national food security. The findings not only confirm the positive role of DT but, more importantly, unpack the “black box” of its underlying mechanisms and identify the spatial boundaries and heterogeneous characteristics of its effects, thereby offering significant insights for deepening our understanding of agricultural transformation in the digital era.

First, the study finds that DT have a significant spatial spillover effect on food security, providing a new perspective for understanding regional agricultural synergy. Traditional agricultural development policies often concentrate on targeted investments in specific regions, such as major grain-producing areas, while overlooking interregional linkage effects. Our findings indicate that the benefits of digital agricultural infrastructure investment in one region are not confined locally but extend to surrounding areas through channels such as technology diffusion and market integration. This means that when formulating digital village strategies, policymakers should transcend administrative boundaries and adopt a perspective of regional integration. For instance, regional agricultural big data centers and DT innovation hubs can be established around central cities, and cross-regional agricultural socialized service platforms and smart logistics networks can be constructed to exert radiating and driving effects on the surrounding rural areas, thereby achieving an overall enhancement of regional food security.

Second, the mechanism analysis underscores the central role of agricultural socialized services in the process of DT empowerment. The mediating effect of socialized services is the largest relative to those of land transfer and climate risk resistance. This suggests that the key to DT-driven agricultural empowerment lies not only in the direct application of technology in the field but also in the technological reshaping of agricultural production organization. Digital platforms can effectively bridge small-scale farmers and modern agricultural services, integrating fragmented and non-standardized production links into specialized and large-scale service systems. This offers a distinct Chinese approach to addressing the questions of “who will farm” and “how to farm well.” Future policies should vigorously support the development of new forms of digital platform-based agricultural socialized services and encourage service organizations to leverage IoT, big data, and other technologies to deliver precise and customized services, thereby effectively integrating the vast number of small-scale farmers into the trajectory of modern agricultural development.

Furthermore, the heterogeneity analysis reveals the conditional nature of DT's enabling effect, which holds important implications for targeted policy implementation. The findings indicate that DT play a more pronounced role in non-major grain-producing areas, flat-terrain areas, and financially developed areas. This does not imply that DT investments in major grain-producing areas and less favorable regions should be neglected. On the contrary, it suggests that policy priorities should be differentiated across regions. For non-major production areas, the potential of DT to achieve leapfrog development should be fully harnessed to vigorously promote technology-intensive agriculture such as urban agriculture and facility agriculture, compensating for insufficient resource endowments. For major grain-producing areas, the policy focus should be on promoting the deep integration of DT with existing mature production systems, for example, using DT to optimize the scheduling efficiency of large-scale agricultural machinery and enhance the intelligence of grain storage and logistics. In regions with complex terrain and underdeveloped financial systems, digital infrastructure development should be coordinated with improvements in traditional infrastructure such as transportation and water conservancy, as well as the establishment of inclusive financial service systems, to create enabling conditions for the effective application of DT and prevent the further widening of the “digital divide”.

Finally, this study has several limitations that point to directions for future research. For instance, the measurement of food security in this study primarily focuses on the “quantity” dimension, namely, per capita output. Future research could develop a more comprehensive food security evaluation framework that incorporates dimensions such as food quality and safety, nutrition and health, and supply chain resilience, and examine the multidimensional impact of DT across these areas. In addition, this paper relies on macro-level data at the prefecture-level city level, which, while capable of revealing overall patterns, cannot provide deep insights into the behavioral decisions of micro-level agents. Future research, combined with large-scale surveys of farmers or new agricultural business entities and employing microeconometric methods, could examine DT adoption behavior and its effects on production efficiency and income in greater detail, thereby complementing and corroborating the macro-level findings of this study.

## 8 Community case analysis

To complement the econometric analysis and illuminate the micro-level mechanisms through which DT enhance food security, this section presents two representative cases from China. These cases serve two primary purposes. First, they offer real-world evidence that corroborates the core theoretical hypotheses developed in Section 3. Second, they demonstrate how a common enabling logic of DT operates across diverse crops, geographical regions, and implementation models. Specifically, the first case (Datong, northern dryland corn) centers on land scale management and agricultural socialized services, corresponding primarily to Hypotheses H1 and H2. The second case (Hubei Maimai, southern citrus) focuses on climate resilience and precision decision-making, corresponding primarily to Hypothesis H3. Both cases further illustrate the positive spatial spillover effect (H4) through technology diffusion and market linkages, albeit at a regional demonstration level. By explicitly linking these cases to the theoretical framework, we seek to bridge the gap between aggregate empirical findings and on-the-ground implementation.

### 8.1 The case of smart agriculture improving corn yield

In 2024, Datong Municipal government organized the implementation of “Beidou + smart agriculture” to support the large-scale maize yield improvement pilot project. The project is located in the east of Xiguzhuang Village, Pingcheng District, Datong city, with a total area of 1,180 mu. The project adheres to the integration of agricultural machinery and agrology, supporting improved varieties and methods, high yield and efficient integration, and implements intelligent, accurate and unmanned management for the whole process of corn production combined with modern information technology.

1. Production problems. The original agricultural production in the project area lacks smart agricultural system and equipment, and information technology and agronomic technology fail to effectively coordinate, which restricts the overall improvement of production benefits. Before the implementation of the project, agricultural production in the region faced double difficulties: (1) at the level of technical equipment, there was a lack of support from smart agricultural system, and digital facilities such as Internet of Things monitoring and intelligent control were blank. (2) At the level of technological coordination, information technology and agronomic technology are separated from each other, which is difficult to form a systematic synergy and restricts the overall improvement of production efficiency. These two problems are universal in the dry farming area of North China, which makes this case have better typification and promotion value.
2. sectionProject plan. The project has built two information systems of “5G+ corn production command” and “5G+ corn production management”, and integrated the application of Beidou unmanned equipment, navigation terminal, weather station, pest and disease monitoring station, soil moisture monitoring station, seedling monitoring instrument and water and fertilizer drip irrigation control equipment and other 7 types of intelligent equipment. (1) Project construction of “5G+ corn production command system”. The system displays land resources, crop distribution and growth information in the form of “a map”. Relying on the Internet of Things equipment to collect data in the pilot area in real time, the system provides a “panoramic view” for analysis and decision-making, realizes unified control, unified scheduling and overall command, and improves the comprehensive management efficiency of agriculture and rural areas in the city. (2) The project established “5G+ corn production management system”. Through the unmanned transformation of existing agricultural machinery, the project can realize the unified scheduling and accurate operation of agricultural machinery in the city. The system supports intelligent task allocation and unified scheduling of agricultural machinery, effectively avoiding idle equipment and improving operation efficiency in busy agricultural seasons. Managers can monitor the progress, quantity and quality of agricultural machinery in real time, providing accurate data support for the supervision of agricultural and rural departments. High-precision navigation and variable control reduce operation deviation to centimeter level, which helps to increase planting density, improve yield per unit area, and reduce harvest loss. Through agricultural machinery management, the whole process from land preparation to harvest can be unmanned, which significantly reduces labor costs and improves operation efficiency.
3. Technological innovation. (1) Application technology of improved varieties. All the pilot fields selected compact dense planting varieties: Xianyu 1483. This variety has the advantages of high yield, wide adaptability, strong stress resistance, compact plant type, tolerance to density and lodging, 127 days of growth, providing the basis for improving the yield per unit area of maize. (2) Precision sowing and dense planting technology. The application of Beidou navigation unmanned driving system to implement precision seeding, using wide and narrow row planting, the specific parameters of row spacing of 80 cm, plant spacing of 18 cm, can effectively increase the number of mu of plants., and finally seeding, fertilization, film covering and pipe laying integrated precise operation. Improve the ventilation and light transmission ability of the group and give full play to the advantages of side walking. The sown density of corn in the pilot area reached 5,997 plants per mu (4,500 plants/mu in the traditional scheme), with a seedling emergence rate of 90.3%, and the number of seedlings protected per mu was 5,421, 7.3 percentage points higher than that in the non-pilot area. This measure highlights the improvement effect of precise navigation technology on land carrying efficiency. (3) Integration of water and fertilizer technology. According to the water and fertilizer demand law of key growth cycles such as maize seedling stage, big horn stage, heading stage and grain filling stage, the project formulated differentiated intelligent irrigation and fertilization scheme, and realized the integrated and accurate application of water and fertilizer relying on the drip irrigation system under film. The monitoring data showed that irrigation water use was reduced by 40% and fertilizer use was reduced by 15%, which verified the optimization effect of the intelligent decision system on resource utilization efficiency. (4) Green prevention and control technology. The project relies on automatic monitoring equipment of diseases and pests to realize real-time perception of diseases and pests, and combines precision drug application of plant protection UAV to grasp the occurrence of diseases and pests in real time. No major pests and diseases occurred in the pilot area in 2024, indicating that the collaborative application of intelligent monitoring and precision medicine application significantly improved the effectiveness of pest and disease prevention and control.
4. Cost effectiveness. According to the experts' on-site yield test, the average yield of corn in the pilot field reached 2,481 jin/mu, 333 jin higher than last year, an increase of 15.5%. The total cost per mu was 1,510 yuan, including 700 yuan for land rent, 95 yuan for seeds, 35 yuan for mulching film, 50 yuan for pesticides, 60 yuan for water, 160 yuan for farm machinery operation, 260 yuan for fertilizer, and 150 yuan for labor. The total revenue per mu is 2,845 yuan, including 2,605 yuan from corn sales, 90 yuan from straw feed and 150 yuan from project subsidies. The input-output ratio is 1:1.88, and the net income is 1,335 yuan/mu.
5. Case experience. The successful practice of Datong pilot project provides three inspirations for understanding the micro implementation mechanism of digital agriculture: (1) the enabling effect of DT depends on the integrated application of technical system, rather than the introduction of single equipment; (2) The coordination of improved varieties, good laws, good opportunities and good systems is the institutional basis for releasing the potential of DT; (3) The application of precise navigation and intelligent decision-making technology can achieve output improvement without increasing resource input, reflecting the intensive characteristics of technological progress.
6. Linkage to theoretical mechanisms. This case clearly illustrates Hypothesis H2 (land transfer efficiency) and Hypothesis H1 (agricultural socialized services). Beidou-enabled precision seeding and dense planting technologies allowed land to be operated at a larger scale (from fragmented plots to 1,180 mu of contiguous pilot area), effectively increasing the number of plants per mu from 4,500 to 5,997. Meanwhile, the “5G + corn production command and management systems” serve as a digital platform for socialized services, coordinating agricultural machinery scheduling, water and fertilizer integration, and pest monitoring. The substantial improvement in yield (a 15.5% increase) and resource efficiency (a 40% reduction in irrigation water and a 15% reduction in fertilizer use) directly supports the theoretical prediction that DT enhance food security by reducing transaction costs in the land market and improving service adoption.

### 8.2 Iot +AI enables accurate decision-making in citrus farming

Hubei Maimai Agricultural Technology Co., Ltd. was established in July 2022. With “digital base + scene enabling” as the core, the company integrates traditional agricultural production with cutting-edge technologies such as Internet of Things, artificial intelligence, cloud computing and blockchain to form a standardized technology package that can be quickly replicated. It adopts the service mode of “science and technology + consumption” and the industrial coordination mechanism of “enterprise + cooperative + farmer”. It provides a replicable and extensible practice path for agricultural modernization transformation. At present, the technology has been extended to 12 provinces and cities including Hubei, Chongqing, Yunnan and Sichuan, enabling more than 300 breeding bases across the country.

1. Production problems. (1) Environmental regulation depends on empirical decision-making. In agricultural production, the monitoring and regulation of key environmental parameters such as temperature and humidity, light and carbon dioxide concentration mainly rely on farmers' experience, and lack accurate quantitative means. Crop growth is easily restricted by natural conditions, and it is difficult to guarantee the stability of yield and quality. (2) Lack of systematic coordination in resource integration. Agricultural production involves multiple factor systems such as soil, meteorology, seedlings, water and fertilizer, and plant protection. (3) There are information barriers to product traceability. The supply chain of agricultural products from the field to the dining table is long and there are many participants. These problems are universal in the vast rural areas and constitute the realistic basis of technological intervention in agricultural science and technology enterprises.
2. Practice methods. The company has established an integrated farmland perception “neural network” and incorporated AI models to enable crop growth simulation, pest and disease prediction, and water and fertilizer optimization. These advances have addressed core challenges in agricultural production, including difficulties in environmental regulation, resource integration, and product traceability, while elevating the level of agricultural precision and intelligence. (1) Precision in production processes. The project leverages an Internet of Things sensor network to collect environmental parameters in real time and, in combination with AI algorithms, achieves automated control of equipment such as irrigation, ventilation, and shading systems. The intelligent pest and disease identification system employs image recognition technology to monitor pest and disease characteristics in real time and generates outbreak trend predictions based on multi-source meteorological data. Prevention and control recommendations are issued 3 to 5 days in advance, and the pest and disease identification rate reaches 95%. Drawing on soil moisture dynamics and crop fertilizer demand patterns, the integrated water and fertilizer system dynamically optimizes the ratio of nitrogen, phosphorus, and potassium (N, P, K) along with irrigation strategies, thereby establishing a fully closed-loop intelligent control system of “perception, decision, and execution”. (2) Scientific management. The company has developed a digital management platform based on multi-source data fusion, integrating remote sensing imagery, UAV field inspection data, and ground sensor data to dynamically optimize planting plans and agricultural management. Through an agricultural knowledge graph and AI-assisted decision-making, resource utilization efficiency is enhanced and labor costs are reduced. The application of blockchain technology enables trusted storage and transparent traceability of information across the entire chain, substantially improving supply chain collaboration efficiency. (3) Software-hardware synergy enabling the entire chain. Centered on its self-developed agricultural digital platform and intelligent hardware, the company deeply integrates advanced equipment, such as DJI unmanned aerial vehicles, to construct an integrated software-hardware solution of “IoT perception plus intelligent decision plus precise execution.” This solution spans the entire chain from production to sales, providing one-stop digital services for cooperative bases. (4) Market-oriented operation. By innovating a service model of “technology plus consumption” and employing the intelligent planting decision-making system as its core driver, the company has built a B2B sales network and formed a full-chain closed-loop ecosystem of “technology improving quality, data optimizing supply, and brand creating premium.” Following the contract farming model of “company plus cooperative plus farmer,” it provides standardized planting technical guidance and comprehensive agricultural input support to cooperatives, along with directed procurement of agricultural products. This ensures both farmers' income growth and raw material quality, and promotes the deep integration of technology and consumption.
3. In terms of technological innovation, Maimai Agricultural Company has constructed a mutually reinforcing three-layer technical system structured around the technical logic of “perception, decision, execution, and traceability”. (1) An integrated “Sky and Land” agricultural sensing and decision-making system. This system comprehensively utilizes satellite remote sensing, UAV multispectral inspection, and ground-based Internet of Things (IoT) sensing devices to achieve multi-dimensional data collection spanning the entire crop growth period and the whole cultivated region. Functioning as “nerve endings,” ground IoT devices transmit 12 types of environmental parameters, including soil temperature, humidity, pH, light intensity, and electrical conductivity (EC), at high frequency, thereby establishing a farmland database with high spatiotemporal resolution. (2) An AI-based crop growth and disaster prediction model. Drawing on intelligent analysis and modeling of massive agricultural data, the system constructs decision-making models for crop growth simulation, pest and disease prediction, and water and fertilizer optimization. Taking the citrus growth model as an example, the system can predict the impact of temperature and humidity variations on fruit set rate during the flowering stage, helping to optimize management strategies. The growth model for Centella centella accurately characterizes the relationship between temperature, light quality, photoperiod, water, and fertilizer and the synthesis of medicinal compounds, thereby enabling a steady increase in the content of key active ingredients. (3) A full-lifecycle traceability system integrating blockchain and IoT. By automatically collecting data on planting, processing, storage, and transportation through IoT devices and employing blockchain technology to achieve tamper-proof storage and trusted data sharing, the system establishes an information closed loop encompassing the entire chain of production, processing, and logistics.
4. Cost effectiveness. (1) Economic benefits. In the 100,000-class high-cleanliness artificial light plant factory project in central Hubei Province, the vegetables produced meet the standard for direct consumption without washing, and the water and fertilizer utilization rate exceeds 75%, representing a 40% saving compared to conventional open-field cultivation. Fully automated management of the entire process reduces labor costs per mu by 50%. Equipped with 80 sets of six-layer three-dimensional cultivation racks, the factory achieves high-density planting of approximately 20,000 plants. With 11 to 12 rapid crop rotations per year, the annual output per unit area reaches 45–50 times that of ordinary open-field cultivation, which typically involves two crops per year. In the Jingmen Zhangfa Citrus Base project, the total annual output value reaches 20 million yuan, the average income per mu increases by approximately 1,000 yuan, the annual benefit grows by over 20%, and the rate of high-quality citrus fruit reaches 85%. Regarding resource conservation, water savings range from 30% to 35%, chemical fertilizer use is reduced by 25% to 28%, pesticide application is cut by 35%, and the annual cost per mu is lowered by more than 400 yuan. Quantitative findings at the technological innovation level further validate the effectiveness of technology empowerment: the application of the AI crop growth model reduced yield fluctuation by 22% and ineffective fertilization by 15%; under the growth model for Centella centella, the total asiaticoside content was steadily increased to over 3.5%, compared to only 0.5% under the conventional cultivation model. The application of the blockchain traceability system increased the added value per unit product by more than 15%. (2) Social benefits. The technology extension activities of Maimai Agricultural Company have generated substantial social benefits. The Jingmen Zhangfa Citrus Base project engaged more than 300 surrounding farmers in smart planting, achieving an average annual income increase of 12,500 yuan per household. The base's associated cooperative absorbed over 200 local laborers and provided technical training for 500 new professional farmers annually, effectively enhancing farmers' digital planting skills and promoting the optimization of the talent structure within the regional citrus industry. This industry coordination mechanism, characterized by the “enterprise + cooperative + farmer” model, offers a replicable pathway for integrating smallholder farmers into modern agricultural development.
5. Case experience. This case study demonstrates that Maimai Agricultural Company has established a four-dimensional enabling pathway encompassing precision production, scientific management, equipment synergy, and market-oriented operation by constructing a three-layer technical framework composed of an integrated “Sky and Land” perception network, an AI crop growth model, and a blockchain traceability system. This framework effectively addresses the core challenges of traditional agricultural production, such as difficulties in environmental control, resource integration, and product traceability. From a theoretical perspective, this case reveals that the core mechanism through which agricultural technology enterprises empower agricultural production lies in substituting data for experience (with real-time perceptual data replacing farmers' experiential judgment), substituting intelligence for manual operations (with AI decision-making and automated control replacing manual work), and substituting systemic integration for fragmentation (with digital integration of the entire chain replacing the decentralized management of separate links). DT have reshaped the production function of traditional agriculture, achieving simultaneous improvements in land productivity, resource utilization efficiency, and labor productivity. From a practical perspective, the “digital base plus scenario enabling” model of Maimai Agricultural Company offers the following values for broader promotion: (1) forming a standardized and rapidly replicable technology package, thereby reducing the cost of technology diffusion; (2) establishing a “technology plus consumption” service model that connects the value chain from production to market; and (3) creating an industrial coordination mechanism of “enterprises plus cooperatives plus farmers” that reconciles technology promotion with farmers' income growth
6. Linkage to theoretical mechanisms. This case primarily illustrates Hypothesis H3 (climate risk resilience) and also reflects Hypothesis H1 (precise socialized services). The IoT sensor network and AI-based crop growth model enabled real-time monitoring of temperature, humidity, soil moisture, and pest conditions, generating early warnings 3 to 5 days in advance and achieving a pest identification rate of 95%. This directly corresponds to the mechanism outlined in Section 3.4, whereby DT reduce perceived climate risk (σ^2^) and enhance production robustness. The reduction in yield fluctuation (a 22% decrease in variability) and the optimization of water and fertilizer use (30 to 35% water savings, 25 to 28% fertilizer reduction) empirically validate H3. Furthermore, the blockchain traceability system and the “company + cooperative + farmer” model embed socialized services (H1) into the value chain, demonstrating how DT can simultaneously address multiple pathways.

### 8.3 Summary of case evidence

Taken together, the two cases corroborate the three primary mediating channels identified in our econometric analysis (Section 6.1). The Datong case underscores land transfer and socialized services, where the marginal effect of DT proved particularly pronounced in a non-major grain-producing area, consistent with the heterogeneity findings reported in Section 6.2. The Hubei Maimai case highlights climate resilience and precision decision-making, demonstrating how DT can stabilize output under conditions of environmental uncertainty. Although the cases differ in crop type (field vs. horticultural) and governance model (government-led vs. enterprise-led), both embody a common underlying logic: the substitution of data for experience, intelligence for manual operation, and systemic integration for fragmentation. These micro-level illustrations complement the city-level spatial econometric findings and provide actionable insights for policy design.

## 9 Conclusions and policy implications

### 9.1 Conclusions

In the face of multiple global risks and challenges, how to take advantage of the opportunities of the DT revolution to ensure national food security is a major issue concerning the national economy and people's livelihood. This paper systematically explores the impact, mechanism of action and spatial effects of DT on food security by constructing theoretical models and conducting empirical analyses using panel data of prefecture-level cities in China. The following main conclusions are drawn from the study:

(1) DT has a significant promoting effect on food security, and this effect has a positive spillover effect in space. The development of DT in a region can not only increase the local per capita grain output, but also, through technology diffusion and market linkage, boost the level of food security in neighboring regions. This conclusion holds true after a series of robustness tests and endogeneity treatments.
(2) The “black box” of DT empowering food security can be explained by three key paths: First, significantly improving agricultural socialized services by reducing information asymmetry; Second, by reducing transaction costs, it effectively promotes land transfer and large-scale agricultural operations; Third, through precise early warning and intelligent management, the resilience of the agricultural system to climate risks has been significantly enhanced. Among these three paths, the mediating effect of agricultural socialized services is most prominent.
(3) There is significant heterogeneity in the enabling effect of DT on food security. The promoting effect is more pronounced in non-grain-producing areas, in flat terrain and in areas with a higher level of financial development, reflecting the inclusive potential of DT and the underlying conditions on which it works.

### 9.2 Policy Implications

Based on the above research findings, this paper presents the following policy implications:

First, the construction of digital villages should be regarded as a new cornerstone for ensuring national food security, and investment in digital infrastructure in rural areas should be continuously increased. Given the significant spatial spillover effect of DT, policy planning should break down administrative barriers and promote the formation of cross-regional agricultural data sharing platforms and smart agriculture demonstration zones to maximize the regional driving effect of DT investment. Particular attention should be paid to the construction of digital infrastructure in the central and western regions and non-major production areas, using DT to bridge the gap in agricultural development between regions.

Secondly, policy support should shift from mere technology promotion to fostering new business forms of “technology + services”. Strong support should be given to the development of agricultural social service organizations based on digital platforms, and they should be encouraged to provide precise and intelligent services throughout the entire industrial chain for small-scale farmers through means such as financial subsidies and tax incentives. Establish and improve digital standards and credit evaluation systems for agricultural socialized services, promote the standardized and healthy development of the service market, and enable DT to truly benefit hundreds of millions of small-scale farmers.

Finally, implement a differentiated and precise digital agriculture development strategy. For regions with different resource access and development stages, policies should be tailored to local conditions. Encourage the development of advanced smart agricultural technologies and create “showrooms” for digital agriculture in areas with flat terrain and developed economies. In areas with complex terrain and underdeveloped economy, the popularization of DT should be combined with the improvement of basic conditions such as transportation and water conservancy and the development of inclusive finance, working together to ensure that DT can take root and be effectively transformed into productive forces that guarantee food security, providing a solid digital support for China's agricultural modernization.

### 9.3 Limitations

(1) Data. Our analysis relies on city aggregate data, which may mask significant within-city heterogeneity between urban centers and rural peripheries. Moreover, the construction of the composite DT index depends on available indicators (AI patents, Internet and mobile penetration rates) due to data availability at the city level. Some emerging DT dimensions, such as blockchain adoption in agriculture, could not be included. The robustness check using a composite food security index is constrained by the availability of city-level data on nutrition, food utilization, and import dependency. (2) Measurement bias. Per capita grain production, our baseline food security indicator, captures only the availability dimension. Although we supplemented with a composite index, the access, utilization, and sustainability dimensions remain imperfectly measured. The proxy for land transfer efficiency (number of agricultural legal entities) is indirect; we acknowledge that the actual transferred area or contract quality would be preferable but are not consistently available across the full sample period. The climate risk resilience indicator simplifies complex climate–agriculture interactions. (3) Causal identification. While we employed an instrumental variable strategy, the exclusion restriction for the IV (historical post offices × national Internet users) may still be violated if historical postal distribution affects current food security through channels other than DT, such as long-term urbanization patterns. We have included historical GDP and urbanization as controls to mitigate this concern, but unobserved historical factors cannot be completely ruled out. Future studies could explore alternative identification strategies, such as quasi-natural experiments from digital infrastructure rollouts.

## Data Availability

Publicly available datasets were analyzed in this study. This data can be found here: https://www.epsnet.com.cn/index.html#/Index.
